# Menthol chewing gum on preoperative thirst management: randomized clinical trial^*^

**DOI:** 10.1590/1518-8345.3070.3180

**Published:** 2019-10-07

**Authors:** Aline Korki Arrabal Garcia, Rejane Kiyomi Furuya, Marilia Ferrari Conchon, Edilaine Giovanini Rossetto, Rosana Aparecida Spadoti Dantas, Ligia Fahl Fonseca

**Affiliations:** 1 Universidade Estadual de Londrina, Londrina, PR, Brasil.; 2 Bolsista da Fundação Araucária de Apoio ao Desenvolvimento Científico e Tecnológico do Estado do Paraná (FA), Brasil.; 3 Bolsista do Conselho Nacional de Desenvolvimento Científico e Tecnológico (CNPq), Brasil.; 4 Instituto Federal do Paraná, Departamento de Enfermagem,Londrina, PR, Brasil.; 5 Universidade de São Paulo,Escola de Enfermagem de Ribeirão Preto, Centro Colaborador da OPAS/OMS para o Desenvolvimento da Pesquisa em Enfermagem,Ribeirão Preto, SP, Brasil.; 6 Universidade Estadual de Londrina, Departamento de Enfermagem, Londrina, PR, Brasil.

**Keywords:** Thirst, Chewing Gum, Menthol, Preoperative Period, Saliva, Mastication, Sede, Goma de Mascar, Mentol, Período Pré-operatório, Saliva, Mastigação, Sed, Goma de Mascar, Mentol, Periodo Preoperatorio, Saliva, Masticación

## Abstract

**Objective:**

to evaluate the effectiveness of menthol chewing gum, in the relief of the intensity and discomfort of the surgical patient’s thirst in the preoperative period.

**Method:**

a randomized controlled trial, with 102 patients in the preoperative period, randomized in a control group, with usual care, and an experimental group, which received menthol gum, which was the study treatment variable. The primary clinical outcome was the variation in thirst intensity, evaluated by the Numeral Verbal Scale, and the secondary, the variation of the discomfort of thirst, evaluated by the Perioperative Thirst Discomfort Scale. Mann-Whitney test was used to compare measures between groups. The significance level adopted was of 0.05.

**Results:**

menthol chewing gum significantly reduced the intensity (p <0.001), with Cohen’s medium-effect d, and thirst discomfort (p <0.001), with a large-effect Cohen’s d.

**Conclusion:**

menthol chewing gum was effective in reducing the intensity and discomfort of preoperative thirst. The strategy proved to be an innovative, feasible and safe option in the use for the surgical patient, in the management of the preoperative thirst, in elective surgeries. NCT: 03200197.

## Abstract (pt)

**Objetivo:**

avaliar a efetividade da goma de mascar mentolada em aliviar a intensidade e o desconforto da sede do paciente cirúrgico no período pré-operatório.

**Método:**

ensaio clínico controlado randomizado, com 102 pacientes em período pré-operatório, aleatorizados em grupo-controle, com cuidado usual, e grupo experimental, que recebeu goma de mascar mentolada, a variável de tratamento do estudo. O desfecho clínico primário foi a variação da intensidade da sede, avaliada pela Escala Verbal Numérica, e o secundário, a variação do desconforto da sede, avaliada pela Escala de Desconforto da Sede Perioperatória. Teste de Mann-Whitney foi usado para comparar as medidas entre os grupos. Nível de significância adotado de 0,05.

**Resultados:**

a goma de mascar mentolada reduziu significativamente a intensidade (p<0,001), com d de Cohen de efeito médio, e o desconforto da sede (p<0,001), com d de Cohen de efeito grande.

**Conclusão:**

a goma de mascar mentolada mostrou-se efetiva na redução da intensidade e do desconforto da sede pré-operatória. A estratégia mostrou-se uma opção inovadora, viável e segura no uso para o paciente cirúrgico, no manejo da sede pré-operatória, em cirurgias eletivas. NCT: 03200197.

## Abstract (es)

**Objetivo:**

evaluar la efectividad de la goma de mascar mentolada en aliviar la intensidad y la incomodidad de la sed del paciente quirúrgico en el período preoperatorio.

**Método:**

ensayo clínico controlado aleatorizado, con 102 pacientes en período preoperatorio, aleatorizados en grupo control, con cuidado usual, y grupo experimental, que recibió goma de mascar mentolada, la variable de tratamiento del estudio. El resultado clínico primario fue la variación de la intensidad de la sed, evaluada por la Escala Verbal Numérica, y el secundario, la variación de la incomodidad de la sed, evaluada por la Escala de Desconocimiento de la Sede Perioperatoria. La prueba de Mann-Whitney fue utilizada para comparar las medidas entre los grupos. Nivel de significancia adoptado de 0,05.

**Resultados:**

la goma de mascar mentolada redujo significativamente la intensidad (p <0,001), con d de Cohen de efecto promedio, y el malestar de la sed (p <0,001), con d de Cohen de efecto grande.

**Conclusión:**

la goma de mascar mentolada se mostró efectiva en la reducción de la intensidad y de la incomodidad de la sed preoperatoria. La estrategia se mostró una opción innovadora, viable y segura en el uso para el paciente quirúrgico, en el manejo de la sed preoperatoria, en cirugías electivas. NCT: 03200197.

## Introduction

Thirst is a present, intense and pre-operative stressor symptom. In this period, the patient is subjected to a series of discomforts during the preparation for the anesthetic-surgical procedure. Emotions such as fear, anxiety and stress trigger physiological reactions, among them, the inhibition of salivary production, causing dryness of the oropharyngeal cavity^(1)^. However, this is not the only challenge the patient faces.

In the preoperative period, as fasting time is prolonged and fluid ingestion is restricted, changes in the electrolyte balance begin to occur^(2)^. Among the physiological responses that occur aiming at its reestablishment, thirst is one of the most relevant, since it acts both in the genesis and cessation of the search for water intake. The thirst resulting from changes in osmolarity and dryness of the oral cavity is considered to be one of the most uncomfortable and stressful experiences for the patient in the perioperative period^(3-5)^. It can be identified by a self-controlling effect, called negative valence^(2,6)^, and is accompanied by the following uncomfortable attributes: dry mouth, lips and throat, thick tongue and saliva, poor taste in the mouth and a desire to drink water^(1,7)^.

The attributes related to the dry mouth, lips and throat increase, exponentially, the discomfort generated by thirst^(1,7)^. Saliva, which has a primordial role in hydration of the mucosa, presents a hydric regulating potential of the body. In situations where the body is deprived of water, dehydration of the oropharyngeal mucosa occurs^(8)^, which leads to the activation of the osmoreceptors, which, in turn, trigger the release, among others, of the antidiuretic hormone (ADH), which acts by preventing water loss, until there is water replenishment. Evidence shows that, in parallel, these osmoreceptors, through afferent pathways, activate the osmosensitive nuclei of the lamina terminalis, which are recognized as responsible for thirst control^(2,6,9)^.

There are two mechanisms of thirst satiety control: the post-absorptive, in which the satiety activation is slower, since the fluid must be absorbed up to of hydroelectrolite balance, and the pre-absortive satiety mechanism, in which the thermoreceptors and oropharyngeal and gastric osmoreceptors are active, which prematurely signal, to the brain, the interruption of ADH release and the consequent thirst sensation^(2)^. Thus, for the surgical patient, the use of strategies that stimulate pre-absorptive satiety is the most adequate, since it occurs even with low volumes.

The use of strategies to relieve the surgical patient’s thirst in the preoperative period is not part of the culture of health institutions, which still coexist with prejudices regarding the administration of any method of postoperative thirst relief. In clinical practice, even delays and surgical suspensions by anesthesiologists and surgeons are recorded, when the patient makes use of chewing gum due to fear of increased gastric contents. However, recent meta-analysis has shown that the use of chewing gums does not increase gastric volume and acidity clinically, significant to the point of triggering bronchi-aspiration^(10)^. The chewing gum acts to increase salivary pH and salivary flow through a combination of gustatory and mechanical stimulation of the salivary glands^(11)^, decreasing dryness of the mouth and the ill effects that this symptom brings.

Additionally, menthol in chewing gum acts on the oropharyngeal receptors called Transient Receptor Potential Melastatin 8 (TRPM 8), present in the nerve endings of the trigeminal and glossopharyngeal nerves, which may be related to satiety due to its anatomical path, with connections with the hypothalamus and somatosensory region in the cortex^(2,9,12)^.

Studies with high level of evidence have evaluated the use of chewing gum in several hospital settings aiming to quench thirst by stimulating salivary production^(13-15)^ and indicate its benefits for the reduction of thirst and xerostomia. However, there is no scientific evidence from well-controlled studies regarding the use of menthol chewing gum to reduce the intensity and discomfort of thirst in the preoperative period, thus pointing to the relevance of this research. In addition, the innovative approach will assist the professionals in the management of thirst, contributing to the increase of the quality in care.

In view of this, the study aims to evaluate the effectiveness of menthol gum in relieving the intensity and discomfort of the surgical patient’s thirst in the preoperative period.

## Method

A randomized controlled clinical trial, with parallel treatments, consisting of two groups: control group (CG), who received usual care, that is, no intervention for the relief of thirst, and experimental group (EG), which received menthol chewing gum.

The recommendations of the Consolidated Standard Protocol Items: Recommendations for Interventional Trials (SPIRIT)^(16)^ were followed for the research protocol, which was submitted to the registry of randomized clinical trials on clinicaltrials.gov of the US National Institutes of Health, obtaining the number NCT03200197. For the elaboration of the study design, the Consolidated Standards of Reporting Trials (CONSORT) were followed^(17)^.

In compliance with the resolution N. 466/12 of the National Health Council, the Research Ethics Committee Involving Human Beings, State University of Londrina, approved the research, with rulling number 1.770.051 and CAAE 59936316.5.0000.5231.

The study took place in the nursing wards of a tertiary level university hospital in the State of Paraná. It is a public institution, with 316 beds, of the Unified Health System (UHS), which performs a monthly average of 640 elective and emergency surgeries.

The study sample consisted of hospitalized patients of both sexes at the selected hospital, submitted to elective surgery and, who met the inclusion criteria.

The inclusion criteria were: elective surgery; ages between 12 and 65 years; not receiving pre-anesthetic medication; oriented in time and space - For this evaluation, the patient should answer five questions of the researcher: what is your name? how old are you?; what is your hometown? what day is today?; is it morning or afternoon? -; present dentition (natural or artificial); fasting for at least three hours; be available for collection at least three hours before the surgical procedure; verbalize thirst spontaneously or, when questioned, with intensity greater than or equal to three in the Verbal Numerical Scale (VNS)^(18)^. The exclusion criteria were: patient with allergy to menthol; restriction to chewing and / or swallowing; presence of nausea, vomiting or pain at the time of approach; chronic xerostomia; chronic kidney patient; impossibility of communication.

The primary clinical outcome of interest was variation in thirst intensity, assessed by VNS^(18)^, which ranges from zero (without thirst) to ten (intense thirst). The secondary clinical outcome was the variation of thirst discomfort, evaluated by the Perioperative Thirst Discomfort Scale (PTDS), which ranges from zero (no discomfort) to 14 (very uncomfortable) and presents seven attributes: dry mouth, dry lips, tongue thick, thick saliva, dry throat, poor taste in the mouth and desire to drink water^(7)^. The PTDS was elaborated and validated to measure the discomfort caused by thirst in the surgical patient, presents a content index of 0.98 and a reliability index of one, internal consistency evaluated by Cronbach’s alpha of 0.91 and inter-observer equivalence of a measure by weighted Kappa coefficient^(7)^. The study’s treatment variable was the use of menthol chewing gum, offered to the patient at least three hours before the anesthetic-surgical procedure.

The randomization of the pilot test and study randomization were performed through a list generated by the Microsoft Office Excel program^®^, with participants randomly distributed in eight blocks with different numbers of participants in each, thus composing the CG (usual care) and EG (menthol chewing gum).

The concealment of the allocation was made using individual opaque envelopes, sequentially numbered externally, containing the group information defined by the random allocation. A professional who had no contact with the main investigator performed the procedure. The opening of the envelopes only occurred after the initial application of the VNS and PTDS scales, in order to guarantee the blinding of the allocation of participants until the intervention.

The data collection used three instruments: a data collecting instrument and the VNS and PTDS scales. The data collecting instrument was submitted to an apparent validation by five judges, specialists in perioperative Nursing and members of the Thirst Study and Research Group (GPS), with demographic (sex and age) and clinical questions (surgical clinic, solid fasting time, fluid fasting time, American Society of Anesthesiologist (ASA) index, use of opioids and anticholinergics).

Due to the lack of similar studies, a pilot test was performed with 40 patients, divided into two groups of 20, which constituted the CG and EG. The data collection period for the pilot test was from november and december 2016, followed all the methodological steps of the clinical trial, and its subjects did not compose the final research sample.

Sample estimation was done based on the pilot study, with a variation of 1.53 in thirst intensity. The significance level considered 5% for the sample calculation, 95% for the confidence interval and 80% for the study power. The calculations indicated a necessary sample of 88 patients, adding 15% of this total to cases of participants’ losses, making a total of 102 patients (51 per group)^(19)^.

The chewing gum of choice for the pilot test was VALDA X^®^, commercially available, and the established intervention time was 20 minutes. However, patients found it difficult to chew on the gum during this whole period of time. With the last five participants who used the product during the pilot test, there was a change in texture of the product in patients’ mouths, representing a possible risk of swallowing small pieces of gum. Therefore, it was necessary to change the product to TRIDENT^®^ mint gum, also available commercially, with composition and weight similar to the product used in the pilot test, but of a firmer consistency.

There was a reduction in intervention time for the study, from twenty to ten minutes, due to the difficulty found in the chewing time during the pilot test. Throughout the intervention period, the researcher remained with the participant, both in the CG and in the EG. There was no change in the data collection protocol.

Data collection was from january to march 2017, following this sequence of procedures:

1. In the preoperative period, all patients who met the eligibility criteria were invited to participate in the study. The consenting adults signed the Free and Informed Consent Term (FICT); underaged participants signed the Term of Assent and their parents or guardians, the FICT;
2. Collection of demographic and clinical data in medical records;
3. Initial evaluation of the intensity of thirst by VNS and the discomfort of thirst by PTDS;
4. Random and hidden allocation, composing the EG and CG groups;
5. Administration of the intervention pertaining the allocated group:
  EG: each received a unit of TRIDENT^®^ mentholated chewing gum, chewing and swallowing the saliva, in natural rhythm for ten minutes.
  CG: each received the usual care performed in the hospitalization units of the institution under study, that is, no intervention was made during ten minutes of follow-up;
6. Final evaluation of the intensity of thirst by VNS and, of the discomfort of thirst by PTDS, after ten minutes of intervention, for both groups

In the CG, because the patients had intense thirst, a menthol chewing gum was offered, after final evaluation, to relieve their thirst.

The statistical analysis procedure was masked, since, before the data was available, the CG was coded in G1 and the EG in G2 to prevent the statistician from distinguishing the group that received the intervention.

For the analysis of the data, non-parametric tests were used, due to the abnormal distribution of the sample evidenced by the Shapiro-Wilk Test. Intensity and discomfort of thirst were considered as a discrete quantitative variables^(20)^.

Mann-Whitney test was used to compare the intensity and discomfort of the initial and final thirst and the variation between the two groups^(20)^. For all comparisons, a significance level of 5% was adopted, with a confidence interval of 95%.

Spearman Correlation Coefficient (ρ), with a confidence interval (CI) of 95%^(20)^, was applied to analyze the correlation between intensity and thirst discomfort variations and the use of chewing gum. The strength of the analysis was based on the effect size of Cohen’s d: small (0.20-0.49), medium (0.50-0.79) or large (0.80-1.29)^(21)^. The analyses were performed using the IBM - SPSS^®^ software (version 20.0).

## Results

**Figure 1:**
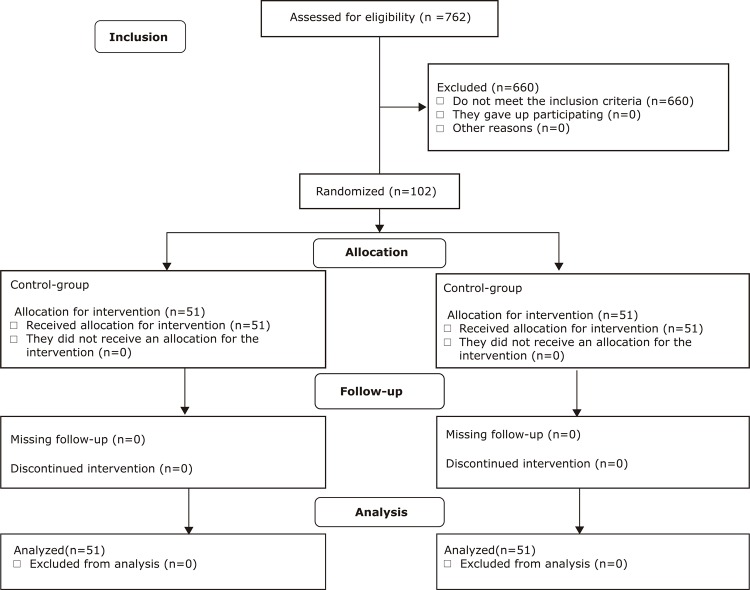
Consort diagram of sampling and randomization. Londrina, PR, Brazil, 2017

During the study period, 762 patients comprised the elective surgical lists. Of these, 547 were out of the eligibility criteria (age, be available for collection at least three hours before the surgical procedure, present clinical conditions). The remaining 215 patients were evaluated for the remaining eligibility criteria (time and space oriented, dentition, minimum fasting of three hours, with thirst with intensity greater than or equal to three by VNS). Eligible patients were invited to participate in the study, thus making up a final sample of 51 patients per group, randomized to CG and EG. There was no loss of segments of participants (Figure 1).

There was no statistically significant difference between groups in relation to demographic and clinical variables prior to randomization (Table 1). The normality test used was the Shapiro-Wilk test, which did not show distribution symmetry. Therefore, the statistical tests used were non-parametric.

**Table 1:** Distribution of demographic and clinical characteristics according to the control and experimental groups. Londrina, PR, Brazil, 2017

Variables	Control-group (n=51) median (± 1^th^-3^rd^quartile)	Experimental group (n=51) median (± 1^th^-3^rd^quartile)	P value*
Age (years)	43.5 (31-49.2)	34.0 (23-41.2)	0.124
Solid fasting (h)^†^	11.94 (10.5-13.2)	12.75 (10.6-15.0)	0.425
Liquid fasting (h)	10.97 (9.4-12.1)	11.08 (10.0-14.2)	0.279

	**n (%)**	**n (%)**	P value*

Sex			
Female	30 (58.8)	29 (56.9)	0.842
Male	21 (41.2)	22 (43.1)	
ASA^‡^			
I	29 (56.9)	36 (70.6)	0.117
II	18 (35.3)	14 (27.5)	
III	4 (7.8)	1 (2.0)	
Opioids			
Yes	16 (31.4)	11 (21.6)	0.264
No	35 (68.6)	40 (78.4)	

*P value = Mann-Whitney test; ^†^h = hours; ^‡^ASA = American Society of Anesthesiologist

When considering the variation in thirst intensity, the EG showed a significant improvement (median = 3) when compared to the CG (median = 0) (<0.001), and Cohen’s d had an average effect (0.77)^(21)^ (Table 2). There was a similar result to that observed in the variation of the discomfort, with the GE obtaining variation (median = 5) and the CG, without (median = 0) (p <0.001), with Cohen’s d with a large effect (0.82)^(21)^ (Table 2).

**Table 2:** Comparison between the control and experimental groups in relation to the intensity and discomfort of the thirst. Londrina, PR, Brazil, 2017

Outcomes	Control-group (n=51) median (± 1^th^-3^rd^quartile)	Experimental Group (n=51) median (± 1^th^-3^rd^quartile)	P value*	d_z_^†^
Initial Intensity	5.0 (4.0-7.0)	6.0 (5.0-6.7)	0.68	-
Final Intensity	5.0 (4.0-7.0)	3.0 (2.0-4.0)	<0.001	0.60
Intensity variation	0.0 (0.0-0.0)	3.0 (1.2-4.7)	<0.001	0.77
Initial discomfort	8.5 (3.75-12.0)	6.5 (3.0-10.7)	0.59	-
Final discomfort	9.5 (3.5-12.7)	1.0 (1.0-2.0)	<0.001	0.79
Discomfort variation	0.0 (-0.7-0.0)	5.0 (1.2-8.0)	<0.001	0.82

*P value = Mann-Whitney test; ^†^d_z_ = d of Cohen extracted from the Z value

In the evaluation of initial discomfort, a high percentage of patients with this symptom was observed in both groups. At the final moment of evaluation, the EG presented improvement, that is, a decrease in the initial values in all the attributes evaluated by PTDS (Table 3).

**Table 3:** Frequency of the attributes of the Perioperative Thirst Discomfort Scale before and after intervention in the control and experimental groups. Londrina, PR, Brazil, 2017

Attributes of PTDS*	Control group		Experimental group	

	Before % (N)	After % (N)	Before % (N)	After % (N)
Dry mouth	64.7 (33)	72.5 (37)	64.7 (33)	3.9 (2)
Dry lips	60.8 (31)	62.7 (32)	58.8 (30)	15.7 (8)
Thick tongue	41.2 (21)	39.2 (20)	49.0 (25)	11.8 (6)
Thick saliva	62.7 (32)	62.7 (32)	47.1 (24)	2.0 (1)
Dry throat	56.9 (29)	60.8 (31)	62.7 (32)	3.9 (2)
Bad taste in the mouth	58.8 (30)	60.8 (31)	49.0 (25)	0.0 (0)
Willingness to drink water	100.0 (51)	98.0 (50)	100.0 (51)	66.7 (34)

^*^PTDS Perioperative Thirst Discomfort Scale

**Figure 2:**
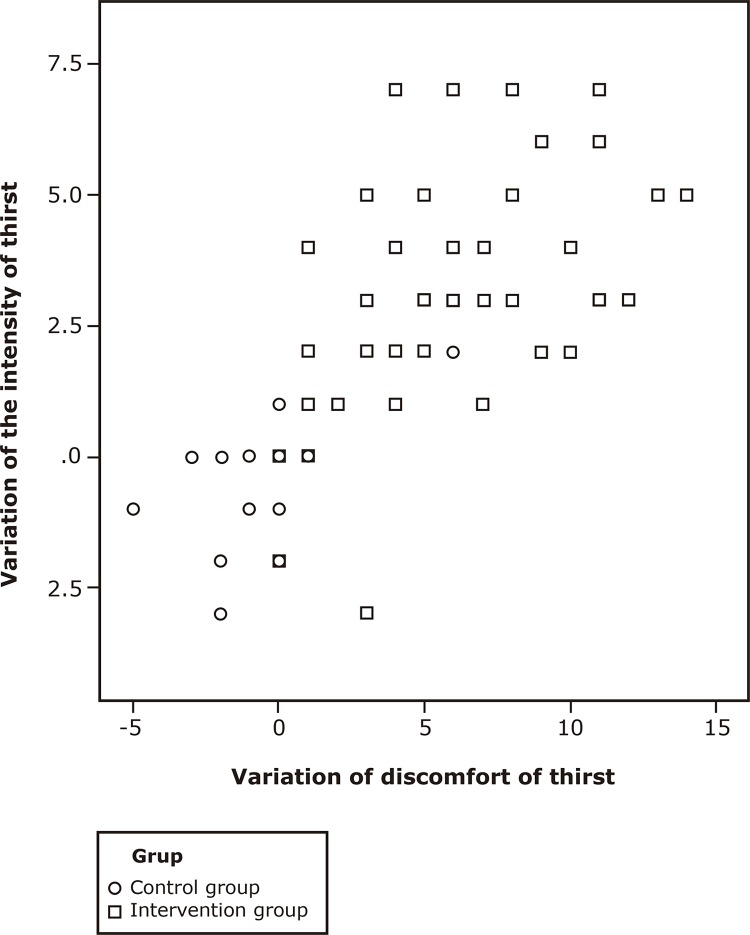
Spearman Correlation Coefficient Scatter plot on the intensity and thirst discomfort variation between groups. Londrina, PR, Brazil, 2017

Spearman’s Correlation showed that the intensity and discomfort variations were positive and strong (ρ = 0.841, p <0.0001) and were related to the use of chewing gum (ρ = 0.778 and 0.831 p <0.0001) (Figure 2).

## Discussion

This study presented an innovative approach for evaluating a simple, feasible, practical, low cost, effective strategy based on physiological mechanisms that act to minimize thirst and its discomforts. In addition, it presents sustained evidences that opose the cultural paradigm that one cannot intervene on preoperative thirst. In addition, in both the pilot and the final study there were no adverse events related to the administration of the chewing gum.

Nonetheless, the team continuously reinforces the impossibility of ingesting any quantity of liquids^(22-23)^. Among the contributions of this research is the finding that the patient in the preoperative also feels thirst. In addition, both the experimental group and the control group presented marked discomfort in relation to thirst at the first moment of evaluation. Menthol chewing gum proved to be effective for the relief of thirst in the preoperative period, considering the medium to large effects found in the intensity variation (Cohen’s d 0.77) and the discomfort (Cohen’s d 0.82) of thirst after the use of a single unit of gum, for a period of only ten minutes. Patients who did not receive the intervention did not present a reduction in thirst.

This data corroborates studies in which there was a similar result in relation to thirst intensity with the use of chewing gum, although conducted with other populations^(13-15)^. Such studies indicate the use of this strategy in patients with xerostomia, in dialysis treatment, also submitted to water restriction^(13-14)^. In addition, chewing gum has also been tested in patients with advanced head and neck cancer who, undergoing radiotherapy, present salivary secretion dysfunctions, leading to oropharynx dryness and therefore thirst^(15)^. The use of the strategy had a positive effect on the stimulation of the salivary glands and consequent increase of salivary flow, reducing thirst^(13-15)^.

The uncomfortable attributes of thirst were identified with high intensity in the preoperative period and are related to salivary decrease and oral dehydration^(1-3,7)^. In this study, the effectiveness of mentholated chewing gum on the discomforts evaluated by PTDS was evidenced. All attributes showed significant reduction after patients received a menthol chewing gum for only ten minutes.

Results highlighted the correlation between the intensity and discomfort variables, as well as the use of mentholated chewing gum, because when one variable was reduced by the use of the strategy, the other presented the same behavior. A study of 203 patients, who evaluated their thirst in the Post Anesthesia Care Unit using PTDS, also found a correlation between intensity and discomfort of thirst^(24)^. This shows that besides evaluating the intensity, it is also important to measure the discomfort related to thirst.

The data showed that the intervention was effective. This positive effect of menthol chewing gum can be explained by three main factors: increased salivary flow through stimulation of the salivary glands, presence of menthol and xylitol in the composition of the gum.

The volume of production of stomach acid secretion in an individual is commonly 0.6 ml.kg^-1^.h^-1^. However, if the same individual remains on a long-term fast, as with surgical patients in the preoperative period, they may present gastric juice production up to 500 ml.h^(25)^. Thus, if the salivary flow rate stimulated by the use of chewing gum is 6.6 ml.min^-1^ in the first minute of chewing, decreasing to 1.5 ml.min^-1^ within 15 minutes^(26-28)^, the use of chewing gum represents a protection factor for the increase of the gastric content.

It is also suggested that preoperative feelings, such as fear, insecurity and anxiety, can generate surgical stress, oral cavity dryness, nausea and hypoglycemia, which stimulate the secretion of ADH and, consequently, sensation of thirst^(5)^. In one study, it was observed that chewing gum can decrease both patients’ anxiety and increase salivary pH^(29)^. In addition, the oral humidification provided by it and increased swallowing of the salivary flow leads to decrease the secretion of ADH^(9)^.

Researches indicate that there is a preference for flavored strategies when compared to paraffin or flavorless chewing gum^(13-15)^. Several studies have used gums flavored with menthol targeting the pleasantness because of the taste, not because of their peculiarity of activating the TRPM 8 receptors, that have a relation with the neural pathways of thirst^(13-15,29)^.

One limitation of the study was the lack of knowledge of the type of menthol that composes the chewing gum used because the chosen gum is commercially available and its formulation is not publicly available. In addition, it was not possible to evaluate the duration of the effect of the menthol strategy on the intensity and discomfort of thirst.

Another factor for the superiority of the intervention is the presence of the sweetener called xylitol, which replaces sucrose in the composition of the gum^(30)^. Among its benefits are the possibility of use by diabetics^(30)^ and its negative value of heat dissolution (-34.8 cal.g^-1^), producing a pleasant cooling effect on the mouth when it comes in contact with saliva. Due to this organoleptic property, xylitol enhances the cooling effect^(30)^ of menthol products such as chewing gum.

The effectiveness of menthol chewing gum in providing a reduction in thirst intensity and discomfort can be explained physiologically, since menthol mimics the action of the cold temperature and activates TRPM8 receptors during gum chewing which decode the presence of menthol in nerve impulses and transmit them through the afferent sensory fibers of the trigeminal and glossopharyngeal nerves. These nerves have their ramifications in the oral cavity, mandible and oropharynx, and their roots are located in the medullary trigeminal nucleus and the nucleus of the solitary tract, respectively, radiating to the supra-optic, paraventricular and subfornical organs, which are highly related areas with stimuli of thirst and secretion of ADH^(2,7,9,31-32)^. The irradiation of these innervations to the anterior cingulate cortex also occurs, more precisely for areas three, two and one of Brodmann, also called somatosensory, which allows the experimentation of distinct sensations, among them, thirst and satiety^(33-35)^.

In view of this, this strategy has high clinical relevance, since its use is simple and feasible in the preoperative period. In addition to being effective, it poses a challenge to the established paradigm in clinical practice regarding surgical suspension in case the patient uses it by his/her own choice^(10)^. Moreover, it is easily applied clinically and represents an increase in the quality of care and humanization due to the intentional look at a basic human need. Moreover this non-pharmacological intervention is low cost and has excellent acceptability by patients^(36)^, who reported a pleasant sensation and intense comfort with the use of the gum.

## Conclusion

There were statistically and clinically significant differences regarding the effectiveness of the menthol chewing gum strategy for the relief of the intensity and discomfort of thirst in the surgical patient in the preoperative period. Given the results evidenced in this study, the conclusion is that this evidence is a simple strategy, of high clinical feasibility, low cost and good patient acceptability. It presents itself as an innovation in the breaking of the paradigm that chewing gum cannot be offered to the surgical patient. It also contributes to the expansion of knowledge in the management of the surgical patient’s thirst, particularly in the preoperative period. It represents an appreciation of nursing care in an individualized way, since it meets a basic human need so commonly neglected.
